# Multimorbidity latent classes in relation to 11-year mortality, risk factors and health-related quality of life in Malaysia: a prospective health and demographic surveillance system study

**DOI:** 10.1186/s12916-024-03796-z

**Published:** 2025-01-06

**Authors:** Michelle M. C. Tan, Charlotte Hanlon, Graciela Muniz-Terrera, Tatiana Benaglia, Roshidi Ismail, Devi Mohan, Ann Breeze Joseph Konkoth, Daniel Reidpath, Pedro José M. Rebello Pinho, Pascale Allotey, Zaid Kassim, Matthew Prina, Tin Tin Su

**Affiliations:** 1 Department of Health Service and Population Research, Institute of Psychiatry, Psychology & Neuroscience (IoPPN), King’s College London, De Crespigny Park, London, UK; 2 Global Public Health, Jeffrey Cheah School of Medicine and Health Sciences, Monash University Malaysia, Subang Jaya, Sunway City, Selangor, Malaysia; 3 South East Asia Community Observatory (SEACO), Monash University Malaysia, Subang Jaya, Sunway City, Selangor, Malaysia; 4 Victorian Heart Institute, Monash University, Victorian Heart Hospital, Clayton Campus, Blackburn Road, Clayton, VIC Australia; 5 Division of Psychiatry, Centre for Clinical Brain Sciences, The University of Edinburgh, Edinburgh, Scotland, UK; 6 Centre for Innovative Drug Development and Therapeutic Trials for Africa (CDT-Africa), Addis Ababa University, Addis Ababa, Ethiopia; 7 Edinburgh Dementia Prevention, The University of Edinburgh and Western General Hospital, Edinburgh, Scotland, UK; 8 Department of Social Medicine, Heritage College of Osteopathic Medicine, Ohio University, Athens, OH USA; 9 Department of Statistics, Institute of Mathematics, Statistics and Scientific Computing, Universidade Estadual de Campinas (UNICAMP), Campinas São Paulo, Brazil; 10 Biostatistics Research Group, Population Health Sciences Institute, Faculty of Medical Sciences, Newcastle University, Newcastle Upon Tyne, UK; 11 Institute for Global Health and Development, Queen Margaret University, Edinburgh, Scotland, UK; 12 Psychogeriatric Unit, Department of Psychiatry, Medical School, Universidade Federal de São Paulo (UNIFESP), São Paulo, Brazil; 13 Department of Sexual and Reproductive Health and Research, World Health Organization (WHO), Geneva, Switzerland; 14 District Health Office Segamat, Ministry of Health Malaysia, Segamat, Johor Malaysia; 15 Population Health Sciences Institute, Faculty of Medical Sciences, Newcastle University, Newcastle Upon Tyne, UK

**Keywords:** Multimorbidity, Latent classes, Health and demographic surveillance system, Mortality, Risk factors, Health-related quality of life

## Abstract

**Background:**

We aimed to identify specific multimorbidity latent classes among multi-ethnic community-dwelling adults aged ≥ 18 years in Malaysia. We further explored the risk factors associated with these patterns and examined the relationships between the multimorbidity patterns and 11-year all-cause mortality risk, as well as health-related quality of life (HRQoL).

**Methods:**

Using data from 18,101 individuals (aged 18–97 years) from the baseline Census 2012, Health Round 2013, and Verbal Autopsies 2012–2023 of the South East Asia Community Observatory (SEACO) health and demographic surveillance system, latent class analysis was performed on 13 chronic health conditions to identify statistically and clinically meaningful groups. Multinomial logistic regression and Cox proportional hazards regression models were conducted to investigate the adjusted association of multimorbidity patterns with the risk factors and mortality, respectively. HRQoL was analyzed by linear contrasts in conjunction with ANCOVA adjusted for baseline confounders.

**Results:**

Four distinct multimorbidity latent classes were identified: (1) relatively healthy (*n* = 10,640); (2) cardiometabolic diseases (*n* = 2428); (3) musculoskeletal, mobility and sensory disorders (*n* = 2391); and (4) complex multimorbidity (a group with more severe multimorbidity with combined profiles of classes 2 and 3) (*n* = 699). Significant variations in associations between socio-demographic characteristics and multimorbidity patterns were discovered, including age, sex, ethnicity, education level, marital status, household monthly income and employment status. The complex multimorbidity group had the lowest HRQoL across all domains compared to other groups (*p* < 0.001), including physical health, psychological, social relationships and environment. This group also exhibited the highest mortality risk over 11 years even after adjustment of confounders (age, sex, ethnicity, education and employment status), with a hazard of death of 1.83 (95% CI 1.44–2.33), followed by the cardiometabolic group (HR 1.42, 95% CI 1.18–1.70) and the musculoskeletal, mobility and sensory disorders group (HR 1.29, 95% CI 1.04–1.59).

**Conclusions:**

Our study advances the understanding of the complexity of multimorbidity and its implications for health outcomes and healthcare delivery. The findings suggest the need for integrated healthcare approaches that account for the clusters of multiple conditions and prioritize the complex multimorbidity cohort. Further longitudinal studies are warranted to explore the underlying mechanisms and evolution of multimorbidity patterns.

**Supplementary Information:**

The online version contains supplementary material available at 10.1186/s12916-024-03796-z.

## Background

Multimorbidity refers to the co-existence of two or more chronic health conditions in an individual [1]. One-third of the population worldwide is living with multimorbidity [2], projected to rise by 2035 [3]. Multimorbidity has become a global public health concern, being associated with higher mortality [4], impaired functional capacity [5], lower quality of life [6], increased healthcare utilization [7] and a greater treatment burden [8]. The impact of synergistic multimorbidity can be much more complex than that of individual diseases, from potential interactions between the diseases, polypharmacy, lifestyle responses and environmental influences. Certain chronic conditions tend to co-occur more often than expected when they share pathophysiological pathways [9]. Identification of patterns of disease combinations and the characteristics of individuals exhibiting similar multimorbidity patterns may offer important information for policymakers and clinicians who seek to integrate strategic public health policy plans and healthcare management to address multimorbidity in higher-risk groups more effectively.

Little is known about multimorbidity patterns in low- and middle-income countries (LMICs), and the body of evidence is even more scarce on the relationships between multimorbidity latent class membership with mortality, risk factors and health-related quality of life (HRQoL) in these settings. Relatively few studies have identified multimorbidity patterns in the general populations of LMICs not restricted to older populations, specific care facilities or patients with specific comorbidities (with a pre-defined index disease), including those in sub-Saharan Africa [10], Jamaica [11, 12], Iran [13], and China [14, 15]. Latent class analysis (LCA) is a data-driven statistical approach that classifies populations into distinct latent classes (also referred to as patterns) or subgroups with similar combinations of chronic health conditions. It is a powerful probabilistic modelling algorithm that permits model-based clustering of data and statistical inference. Unlike other methods of clustering (e.g. cluster analysis, k-means clustering and exploratory factor analysis (EFA)), LCA allows objective testing of model fit and is considered a more statistically robust method [16]. This approach is valuable in healthcare as it models ‘real-world’ scenarios by considering actual combinations of multimorbidity and classifying them into groups that are both statistically valid and clinically interpretable. Moreover, LCA affords greater flexibility by allowing diseases to have partial membership across multiple clusters than other more limiting distance-based clustering methods. Techniques such as EFA require data to be in a continuous format, which is unrealistic for diagnostic variables [9]. Therefore, there has been a recent upsurge in the application and recommendation of LCA in the field of medicine and beyond [16]. Although LCA has been performed to evaluate multimorbidity in various populations, particularly in high-income countries (HICs), including the USA [17] and the UK [18], to our knowledge, LCA has not been applied to examine the multimorbidity combination types and their relationship to health outcomes, including mortality in community-residing general populations in Malaysia and South East Asia.

The key challenges in multimorbidity research stem from existing healthcare systems that are often configured vertically, around single diseases, leading to fragmented care for patients with multimorbidity. Implementing and sustaining interventions for multimorbidity can be costly, and resource allocation may be more challenging, particularly in resource-constrained healthcare systems, such as LMICs. In addition, research that considers both disease-specific and overall health-related outcomes remains limited. This is mainly because collecting and analyzing longitudinal data to understand the long-term effects of multimorbidity patterns and health outcomes, such as mortality tracking, is resource-intensive and requires innovative data collection and analysis methods. Recruiting a representative sample of people with multimorbidity for research, particularly patterns of multimorbidity further limits the available relevant studies, in that a large sample size often is needed for adequate statistical power. A recent systematic review pointed out that studies of multimorbidity patterns in younger populations are clearly under-researched [19], even though multimorbidity is not restricted to older people. To date, no such evidence is available from general populations in Malaysia and South East Asia. Understanding multimorbidity latent classes and how they relate to mortality, higher risk, and HRQoL can provide valuable insights for healthcare planning, resource allocation, and improving the well-being of vulnerable populations with multimorbidity.

Therefore, we aimed to address these existing challenges by identifying specific multimorbidity latent classes using LCA and exploring how these multimorbidity patterns were associated with (a) 11-year all-cause mortality; (b) socio-demographic characteristics; and (c) HRQoL in a large community-dwelling multi-ethnic cohort from prospective health and demographic surveillance system datasets.

## Methods

### Data and sample

**Fig. 1:**
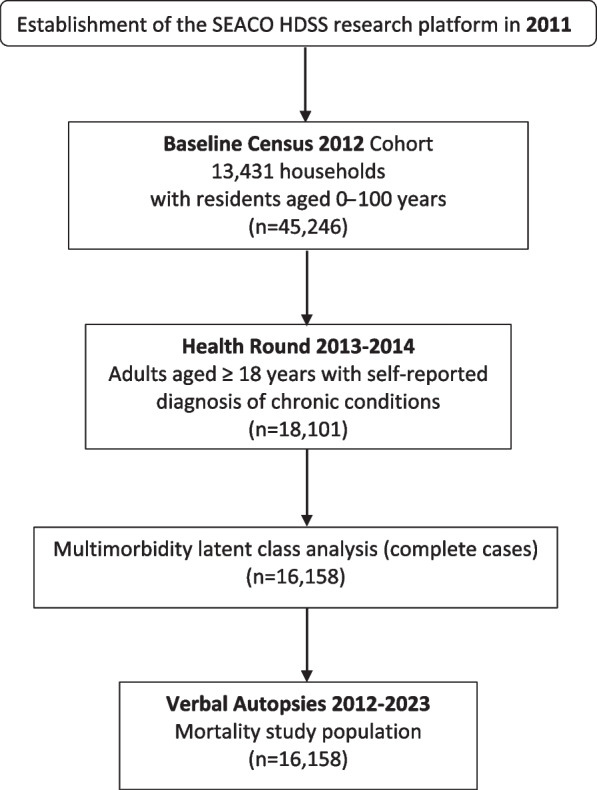
Study sampling flowchart

This study used data from the baseline Census 2012, Health Round 2013 and Verbal Autopsies 2012–2023 of the South East Asia Community Observatory (SEACO) health and demographic surveillance system (HDSS) research platform, established in 2011 in Malaysia. The cohort profile has been described in detail previously [20]. Briefly, the baseline Census 2012 consisted of 45,246 community-dwelling residents aged 0─100 from 13,431 households in predominantly semi-rural areas. In the 2013 “Health Round”, detailed medical history was assessed among 25,168 residents aged 5 years and above who were part of the core sample. Of this sample, 18,101 adults were aged 18 and older [mean age 47.3 years (SD 16.3 years)] and were considered in the present study. The mortality status of the participants was obtained from all Verbal Autopsy rounds and gathered up to 11 years after the Health Round 2013 assessments. The Verbal Autopsy registry containing details of deaths within the community was compiled using information gathered from multiple sources. This includes the ongoing annual enumeration updates and fortnightly updates from key community informants (e.g. SEACO community engagement committees, village leaders, police stations, district health office and district hospitals). Other deaths were captured when informed by family members of participants. Households in which deaths occurred were also approached with consent, at culturally appropriate and convenient dates, for verbal autopsies. These were performed using the World Health Organization (WHO) 2012 verbal autopsy instrument, adapted according to the local context, in consultation with the Ministry of Health [21]. The detailed study population selection is illustrated in Fig. 1.

### Measures of multimorbidity

Presence or absence of a chronic health condition was based on self-reported diagnosis (1 = presence; 0 = absence) on whether participants had ever been informed by a medical professional that they had a particular chronic health condition on a prespecified list and still suffered from effects of the condition. The health conditions were heart disease (including coronary artery disease, myocardial infarction, angina pectoris, congestive heart failure), stroke, type 1 and/or type 2 diabetes mellitus (DM), hypertension, chronic kidney disease, end-stage renal failure (ESRF) requiring dialysis, arthritis, joint pain, back pain, obesity, asthma, vision problems, hearing problems, physical mobility problems and depressive symptoms. Obesity (body mass index (BMI) ≥ 30 kg/m^2^) was defined using the WHO classification [22], calculated as weight (in kilogrammes) divided by height squared (in metres). Some of the health conditions were combined into one condition category conceptually. For instance, joint pain and back pain were combined into ‘chronic pain’. Chronic kidney disease and ESRF requiring dialysis were considered together as ‘chronic kidney disease’. Mental health was measured using the Depression, Anxiety and Stress Scale–21 (DASS-21) [23], for which a cutoff score of ≥ 10 on the depression scale was classified as having depressive symptoms [24]. Hence, the total number of chronic health conditions included in our analysis was 13. Multimorbidity was defined as the presence of two or more of these concurrent chronic physical or mental health conditions for each participant.

### Risk factors

Drawing on evidence from the literature, socio-demographic factors were considered potential risk factors associated with the multimorbidity latent classes. The following socio-demographic characteristics were included in the present study: age, sex (female, male), ethnicity (Malay, Chinese, Indian), education attainment (no formal education, primary, secondary, tertiary education), employment status (working/self-employed, homemaker, unemployed, retired/pension, student), marital status (never married, widowed/divorced/separated, married), and household monthly income (Bottom 40%, Middle 40%, Top 20%).

### Outcome measures

#### Mortality

We assessed all-cause mortality status by following up until the earliest date of death or end of the verbal autopsy interviews in June 2023, whichever came first.

#### Health-related quality of life

Health-related quality of life (HRQoL) of the study population at baseline was measured with the brief version of the World Health Organization Quality of Life (WHOQOL) instrument, known as WHOQOL-BREF [25]. The WHOQOL-BREF is a validated 26-item instrument that assesses the overall quality of life in four domains: physical health, psychological, social and environmental. The response to each item was given on a 5-point Likert score, scaled in a positive direction with higher scores indicating a better quality of life (1 = impossible, 2 = a little, 3 = general, 4 = strong, 5 = completely). The mean score of items within each domain was used to calculate the domain scores. Mean scores were then multiplied by 4 and linearly transformed to make domain scores comparable to scores used in the WHOQOL-100 version.

### Statistical analysis

The data analysis in the present study comprised four steps: (1) identifying latent classes with different disease patterns in the study cohort; (2) analyzing variations in HRQoL across latent classes; (3) examining the association between socio-demographic factors and latent class membership; and (4) evaluating the association between mortality and latent class membership.

First, based on the 13 health conditions, latent classes of multimorbidity were identified using the latent class analysis (LCA) method on complete case data without any a priori assumptions about the number of latent classes. LCA is applied to derive groups of individuals that are relatively homogeneous within a distinct population, assuming that the performance of an individual on a set of items is explained by a categorical latent variable with *k* (where *k* is the number of classes), commonly called ‘latent classes’. In this study, the optimal number of latent classes was guided by the lowest Consistent Akaike Information Criterion (CAIC) and the Bayesian–Schwarz Information Criterion (BIC), clinical significance and interpretability. Entropy statistics and various tests including log-likelihood, chi-squared and likelihood ratio tests were also taken into consideration. Lower log-likelihood, AIC, BIC, chi-squared and likelihood ratio values indicate better-fitted model. An entropy of 0.8 or greater denotes acceptable class separation [26]. The posterior probabilities of membership of each latent class identified were also estimated, and participants were allocated to the classes for which they had the highest posterior probability. We used a recommended probability cutoff of ≥ 0.5 to indicate clear allocation for each latent class [16]. To visualize the classification robustness after fitting the model, dot plots with the posterior probabilities of each observation were generated.

The model was fitted using the *poLCA* package from R software [27], including the 13 health conditions as observed indicators. After the selection of an optimal model, each participant was assigned to the class that had the highest estimated posterior probability of class membership, i.e. the latent class they most likely belonged to. The characteristics of participants in different latent classes were compared using the chi-squared (*χ*^2^) test for categorical variables and one-way analysis of variance (ANOVA) for continuous variables. Continuous variables were expressed as mean ± standard deviation (SD). Categorical variables were expressed as numbers (with percentages). Additionally, we investigated if HRQoL varied by latent class membership using linear contrasts in conjunction with analysis of covariance (ANCOVA) adjusted for possible confounders (age, sex, ethnicity, education, marital status and employment status), implemented by the STATA command *lincom*. The mean of the WHOQOL-BREF index score alongside 95% CI was estimated for each latent class. Based on the lowest WHOQOL-BREF index scores of the complex multimorbidity group identified in ANCOVA, linear contrasts combining the first three latent classes minus the WHOQOL-BREF index scores of the complex multimorbidity group were further constructed to assess the absolute differences in HRQoL between the two newly derived groups.

After identifying latent classes, multinomial logistic regression was performed to assess the association between each socio-demographic factor (age, sex, ethnicity, education, marital status, household monthly income and employment status) and latent class membership. Associations were assessed using adjusted-odds ratios (adjusted-ORs) with 95% confidence intervals (CIs).

Subsequently, to explore the association between multimorbidity patterns and cumulative 11-year all-cause mortality, a Cox proportional hazards regression model, Kaplan–Meier survival curves and a log-rank test were used. Schoenfeld residuals were used to test the proportional hazards assumption in the Cox proportional hazards regression model. The Cox proportional hazards regression model is a method for analyzing the time to the occurrence of an event (in this case, death) based on longitudinal cohort data. In the present study, participants were followed up from the time of the interview in 2012 (start date) to the earliest year of death up to 2023 (end-date). The time to death was calculated as the difference between the year of death and the year of the baseline interview. Mortality risk, expressed as hazard ratios (HRs) adjusted for confounding variables (age, sex, ethnicity, education and employment status), was compared between different groups of individuals based on their multimorbidity patterns. We used the ‘relatively healthy’ group as the reference, as it was the lowest-risk and largest-sized group.

Statistical analyses were performed using R (version 4.3.0, R Foundation for Statistical Computing, Vienna, Austria), Stata/MP (Version 18, Stata Corp LP, College Station, TX, USA) and IBM SPSS Statistics (Version 28, IBM, Armonk, New York, USA). All tests were two-tailed, and a *p*-value < 0.05 was regarded as statistically significant.

## Results

### Characteristics of the study population

Table 1 presents the baseline characteristics of the overall study population residing in the semi-rural setting. Of the 18,101 eligible participants aged 18 to 97 years, over half (55.6%) were female. Two-thirds of the participants were of Malay ethnicity (65.7%), with the majority having attended primary and secondary schools (86.2%) and were of the lowest bottom household monthly income group (75.0%). Only half of them were working (50.3%), and nearly one-third (30.8%) were homemakers.

**Table 1:** Overall study population characteristics

	**Overall (***n* **= 18,101)**
Age (years)	
Mean±SD	47.3±16.3
Range	18–97
Sex, n (%)	
Female	9089 (55.6%)
Male	7268 (44.4%)
Ethnicity, n (%)	
Malay	10,741 (65.7%)
Chinese	3586 (21.9%)
Indian	1594 (9.8%)
Other	418 (2.6%)
Marital status, n (%)	
Never married	2584 (16.0%)
Married	11,846 (73.2%)
Widowed/Divorced/Separated	1745 (10.8%)
Education level, n (%)	
No formal education	841 (5.2%)
Primary	4842 (30.1%)
Secondary	9012 (56.1%)
Tertiary	1373 (8.5%)
Employment status, n (%)	
Working/Self-employed	8189 (50.3%)
Homemaker	5017 (30.8%)
Unemployed	1868 (11.5%)
Retired/Pension	885 (5.4%)
Student	313 (1.9%)
Personal gross monthly income (MYR)	
Mean±SD	1204.0±1515.4
< 1000	8554 (52.3%)
1000-1999	4575 (28.0%)
≥ 2000	3228 (19.7%)
Household monthly income (MYR)^a^	
Mean±SD	3193.8±3537.1
Bottom 40% (B40)	12,263 (75.0%)
Middle 40% (M40)	3076 (18.8%)
Top 20% (T20)	1018 (6.2%)

Abbreviations: *SD* Standard deviation

MYR Malaysian Ringgit, MYR 1 was equivalent to the British Pound (GBP) £ 0.17 at the time of publication

^a^Categorized as three income thresholds according to the local Department of Statistics, where B40, M40 and T20 represent groups with monthly income of < MYR 3860, MYR 3860–8319 and > MYR 8319, respectively

### Multimorbidity patterns and outcomes

Table 2 presents log-likelihood, AIC, BIC, chi-squared test, likelihood ratio and entropy values of the six latent class models performed in 16,158 complete cases. Model fit statistics reveal that most metrics improved as the number of latent classes increased, particularly from three-class model onwards, with entropy values remaining substantial across the three to six latent class models. The chi-squared test value was notably lower for the four-class model, indicating it was significantly better than the three-class model. Although the five-class and six-class models showed slight improvements in fit metrics compared to the four-class model, they detracted from clinical interpretability and relevance. In the four-class model, the latent class 4, which combines the highest number, types, and severity of chronic conditions, enables meaningful comparisons with other classes. Based on these considerations, the four-class model with a better statistical fit and the most reasonable clinical interpretability was selected as the optimal solution among the latent class models.

**Table 2:** A comparison of the fit indices between one to six latent class models

Number of classes	Log-likelihood	AIC	BIC	Chi-squared	Likelihood ratio	Entropy
1	− 58,562.4	11,7150.9	117,250.8	178,900,003.8	9642.8	3.6
2	− 56,180.7	112,415.4	112,623.0	130,941.0	4878.8	3.5
3	− 55,091.6	110,265.2	110,580.5	165,767.1	2700.5	3.4
4	− 54,915.5	109,940.9	110,363.9	38,651.8	2348.2	3.4
5	− 54,828.0	109,794.0	110,324.7	27,418.8	2173.3	3.4
6	− 54,753.0	109,671.9	110,310.2	16,999.8	2023.2	3.4

Abbreviations: *AIC* Akaike Information Criteria, *BIC *Bayesian Information Criterion

**Fig. 2:**
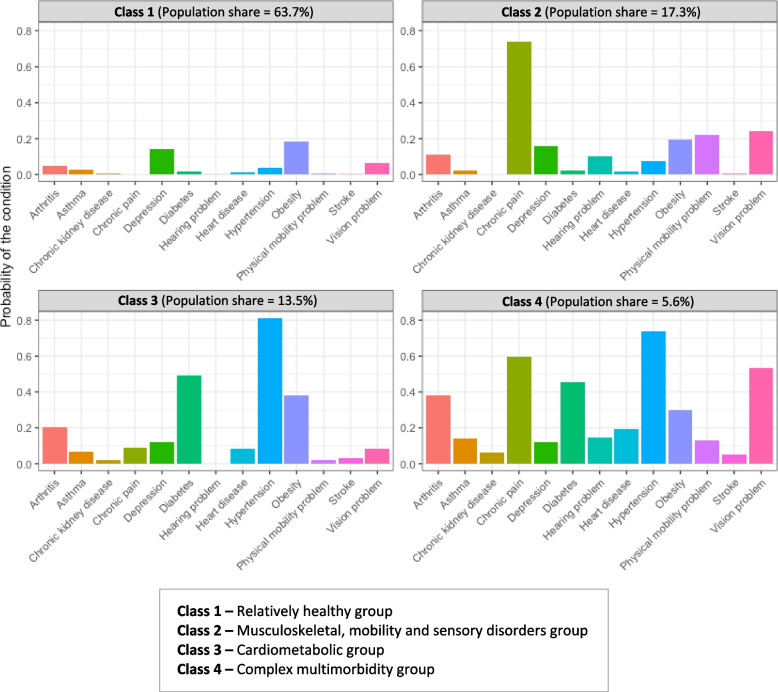
Graphical representation of the four latent classes by the probability of class membership of the chronic health conditions (*n *= 16,158)

Figure 2 illustrates the four resultant classes by the probability of class membership of the chronic health conditions, represented by population shares of 63.7%, 17.3%, 13.5% and 5.6%, respectively, among the classes. We labelled the four derived classes according to the greatest predicted probability of chronic conditions in each class. The four patterns of multimorbidity identified were (a) *relatively healthy group (latent class 1)* (*n* = 10,640) composed of little to no chronic conditions compared to the total sample, (b) *musculoskeletal, mobility and sensory disorders group (latent class 2)* (*n* = 2391) with the highest prevalence of arthritis, chronic pain and a range of physical health conditions including physical mobility, hearing and vision problems, (c) *cardiometabolic group (latent class 3)* was driven by heart disease, stroke, hypertension, diabetes and obesity (*n* = 2428) and (d) *complex multimorbidity group (latent class 4)* corresponding to a more severe group with multiple overlapping morbidities (*n* = 699). Depressive symptoms were of a similar level across the latent classes and, as such, were not classified into a specific class. The prevalence of all chronic conditions in all classes are provided in Table 3.

**Table 3:** Population characteristics stratified by the multimorbidity latent classes (*n *= 16,158)

Characteristics	Latent class				*p*-value
	Class 1 (Relatively healthy) (*n* = 10,640)	Class 2 (Musculoskeletal, mobility and sensory disorders) (*n* = 2391)	Class 3 (Cardiometabolic) (*n* = 2428)	Class 4 (Complex multimorbidity) (*n* = 699)	
**Socio-demographics**					
Age (years)					
Mean±SD	43.1±15.5	50.8±16.3	57.3±11.1	62.4±11.6	<0.001
n (%)					<0.001
18-34	3577 (33.6%)	461 (19.3%)	70 (2.9%)	12 (1.7%)	
35-59	5376 (50.5%)	1165 (48.7%)	1334 (54.9%)	254 (36.3%)	
60 and above	1687 (15.9%)	765 (32.0%)	1024 (42.2%)	433 (61.9%)	
Sex, n (%)					<0.001
Female	5739 (53.9%)	1405 (58.8%)	1435 (59.1%)	406 (58.1%)	
Male	4901 (46.1%)	986 (41.2%)	993 (40.9%)	293 (41.9%)	
Ethnicity, n (%)					<0.001
Malay	7055 (66.4%)	1500 (62.8%)	1615 (66.6%)	456 (65.2%)	
Chinese	2212 (20.8%)	662 (27.7%)	498 (20.5%)	156 (22.3%)	
Indian	1077 (10.1%)	181 (7.6%)	250 (10.3%)	65 (9.3%)	
Other	281 (2.6%)	47 (2.0%)	63 (2.6%)	22 (3.1%)	
Marital status, n (%)					<0.001
Never married	2131 (20.3%)	313 (13.2%)	70 (2.9%)	28 (4.0%)	
Married	7552 (72.0%)	1705 (71.8%)	1941 (80.1%)	510 (73.1%)	
Widowed/Divorced/Separated	802 (7.6%)	355 (15.0%)	412 (17.0%)	160 (22.9%)	
Education level, n (%)					<0.001
No formal education	448 (4.3%)	250 (10.7%)	79 (3.3%)	51 (7.4%)	
Primary	3115 (29.8%)	923 (39.7%)	565 (23.6%)	161 (23.5%)	
Secondary	6005 (57.4%)	1002 (43.1%)	1511 (63.2%)	399 (58.2%)	
Tertiary	901 (8.6%)	152 (6.5%)	235 (9.8%)	74 (10.8%)	
Employment status, n (%)					<0.001
Working/Self-employed	5925 (56.0%)	1124 (47.4%)	857 (35.4%)	209 (30.0%)	
Homemaker	3037 (28.7%)	750 (31.7%)	931 (38.4%)	239 (34.3%)	
Unemployed	997 (9.4%)	278 (11.7%)	398 (16.4%)	165 (23.7%)	
Retired/Pension	372 (3.5%)	170 (7.2%)	235 (9.7%)	83 (11.9%)	
Student	255 (2.4%)	47 (2.0%)	3 (0.1%)	1 (0.1%)	
Household monthly income (MYR)^a^					
Mean±SD	3290.4±3424.7	2901.8±3427.3	2672.9±3147.0	2093.2±2644.6	<0.001
n (%)					<0.001
Bottom 40% (B40)	7718 (72.5%)	1841 (77.0%)	1938 (79.8%)	597 (85.4%)	
Middle 40% (M40)	2186 (20.5%)	406 (17.0%)	374 (15.4%)	85 (12.2%)	
Top 20% (T20)	736 (6.9%)	144 (6.0%)	116 (4.8%)	17 (2.4%)	
**Clinical**					
Chronic conditions, n (%)					
Arthritis	552 (5.2%)	260 (10.9%)	425 (17.5%)	330 (47.2%)	<0.001
Asthma	307 (2.9%)	56 (2.3%)	143 (5.9%)	115 (16.5%)	<0.001
Chronic kidney disease	49 (0.5%)	0 (0.0%)	44 (1.8%)	53 (7.6%)	<0.001
Chronic pain	0 (0.0%)	2135 (89.3%)	137 (5.6%)	522 (74.7%)	<0.001
Depressive symptoms	1563 (14.7%)	387 (16.2%)	239 (9.8%)	77 (11.0%)	<0.001
Diabetes mellitus	197 (1.9%)	56 (2.3%)	1107 (45.6%)	338 (48.4%)	<0.001
Hearing problem	0 (0.0%)	295 (12.3%)	0 (0.0%)	131 (18.7%)	<0.001
Heart disease	117 (1.1%)	35 (1.5%)	178 (7.3%)	165 (23.6%)	<0.001
Hypertension	56 (0.5%)	137 (5.7%)	2253 (92.8%)	586 (83.8%)	<0.001
Obesity	1945 (18.3%)	443 (18.5%)	933 (38.4%)	207 (29.6%)	<0.001
Physical mobility problem	1 (0.0%)	653 (27.3%)	48 (2.0%)	106 (15.2%)	<0.001
Stroke	29 (0.3%)	17 (0.7%)	67 (2.8%)	41 (5.9%)	<0.001
Vision problem	765 (7.2%)	543 (22.7%)	210 (8.6%)	452 (64.7%)	<0.001
Number of chronic conditions					<0.001
Mean±SD	0.5±0.7	2.1±0.9	2.4±1.1	4.5±1.2	
**Quality of life**^b^					
WHOQOL-BREF index score, Mean (95% CI)					
Physical health domain	60.6 (60.4–60.8)	60.1 (59.6–60.6)	60.6 (60.1–61.1)	58.3 (57.4–59.2)	<0.001
Psychological domain	61.4 (61.2–61.7)	61.5 (61.0–62.0)	61.7 (61.2–62.2)	59.2 (58.3–60.2)	<0.001
Social relationship domain	70.5 (70.2–70.8)	70.2 (69.6–70.8)	71.0 (70.4–71.7)	67.6 (66.5–68.7)	<0.001
Environment domain	65.9 (65.7–66.2)	65.8 (65.2–66.3)	66.0 (65.5–66.5)	64.1 (63.1–65.1)	0.004

Abbreviations: *SD* Standard deviation, *WHOQOL-BREF *World Health Organization Quality of Life Brief Version, *CI *Confidence interval

MYR = Malaysian Ringgit, MYR 1 was equivalent to the British Pound (GBP) £ 0.17 at the time of publication

Numbers may not add up to totals due to missing data

^a^Categorized as three income thresholds according to the local Department of Statistics, where B40, M40 and T20 represent groups with monthly income of < MYR 3860, MYR 3860–8319 and > MYR 8319, respectively

Statistical significance testing by chi-square (*χ*^2^) test and one-way ANOVA with post-hoc Bonferroni test unless otherwise indicated

^b^Analysis of covariance (ANCOVA) adjusted for age, sex, ethnicity, education, marital status and employment status

*p<0.05, **p<0.01, ***p<0.001

**Fig. 3:**
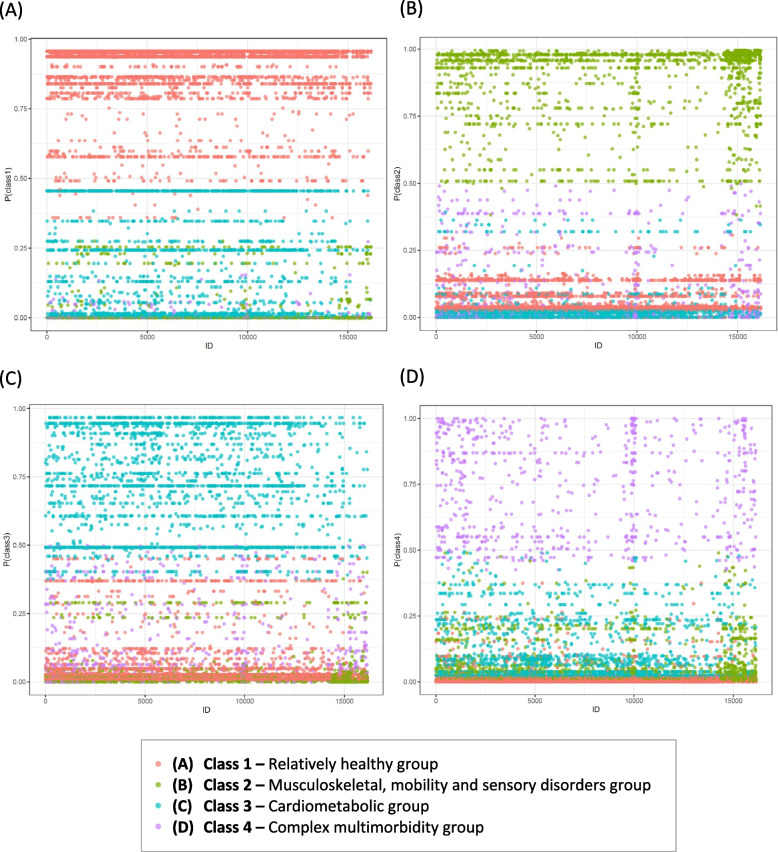
Dot plots visualizing the posterior probabilities of membership for each identified latent class (y-axis) across all participants (x-axis). Red dots indicate participants classified into Class 1, green dots represent Class 2, blue dots correspond to Class 3, and purple dots signify Class 4. The vertical position of each dot reflects the probability value, ranging from 0 to 1.0. A probability close to 1.0 for a particular class and close to 0 for the others indicates a robust classification

Figure 3 A─D graphically presents the posterior probabilities for the four-class models. Each dot represents the study participant's probability of belonging to a respective latent class, with the colour indicating the assigned class. Our plots, with a high concentration of dots near a probability of 1.0 and greater than 0.5, indicate that participants classified into classes 1 through 4 were likely to be accurately categorized. There were high average posterior probabilities for all the four classes ─ 0.9 for both the relatively healthy group and the musculoskeletal, mobility and sensory disorders group, and 0.7 for both the cardiometabolic group and the complex multimorbidity group.

The distribution of the study population characteristics segregated by the four latent classes is depicted in Table 3. All the socio-demographic and clinical characteristics of the participants differed significantly across the latent classes. Among the latent classes, latent class 1 (relatively healthy group) tended to be the youngest, with a mean age of 43.1 years, whereas latent class 4 (complex multimorbidity) had the highest mean age population (62.4 years). The most prevalent chronic conditions were obesity (21.8%), hypertension (18.8%) and chronic pain (17.3%).

HRQoL scores were consistent in the first three latent classes, with a marked distinction in the fourth latent class (complex multimorbidity group), which was much lower (Table 3). Based on linear contrasts in conjunction with ANCOVA, being in the complex multimorbidity group evidenced a significantly lower HRQoL than other groups across the four domains: physical health domain (2.1 lower WHOQOL-BREF index score, *p* < 0.001, 95% CI = 1.2–3.1), psychological domain (2.3, *p* < 0.001, 95% CI = 1.4–3.2), social relationship domain (2.9, *p* < 0.001, 95% CI = 1.7–4.1) and environment domain (1.8, *p* < 0.001, 95% CI = 0.8–2.8).

#### Factors associated with multimorbidity patterns

Table 4 presents the results of multinomial logistic regression for the four-class model, with latent class 1 (relatively healthy group) set as the reference group. Compared to the relatively healthy group, older age consistently raised the probability of being assigned to the musculoskeletal, mobility and sensory disorders group (adjusted-OR = 1.04, 95% CI = 1.03–1.04), cardiometabolic group (adjusted-OR = 1.06, 95% CI = 1.06–1.06) and complex multimorbidity group (adjusted-OR = 1.10, 95% CI = 1.09–1.10). Being male significantly decreased the likelihood of belonging to the musculoskeletal, mobility and sensory disorders (adjusted-OR = 0.78, 95% CI = 0.69–0.88), but not the cardiometabolic and complex multimorbidity groups. Being Malay (adjusted-OR = 1.32, 95% CI = 1.11–1.57) and Chinese (adjusted-OR = 1.69, 95% CI = 1.40–2.03) were more likely than Indian to belong to the musculoskeletal, mobility and sensory disorders group among the study population, but not the cardiometabolic and complex multimorbidity groups.

**Table 4:** Multinomial logistic regression modelling results of factors associated with multimorbidity patterns

Parameter	**Latent Class**					
	Musculoskeletal, mobility and sensory disorders group		Cardiometabolic group		Complex multimorbidity group	
	Adjusted-OR	95% CI	Adjusted-OR	95% CI	Adjusted-OR	95% CI
Age (years)	1.04***	1.03–1.04	1.06***	1.06–1.06	1.10***	1.09–1.10
Sex						
Male	0.78***	0.69–0.88	0.89	0.78–1.01	0.90	0.72–1.11
Female (Ref)	1		1		1	
Ethnicity						
Malay	1.32**	1.11–1.57	1.02	0.87–1.19	1.12	0.85–1.49
Chinese	1.69***	1.40–2.03	1.09	0.91–1.31	1.32	0.97–1.81
Indian (Ref)	1		1		1	
Education						
No formal education	3.64***	2.85–4.65	0.69*	0.51–0.95	1.60*	1.06–2.40
Primary	1.90***	1.57–2.31	0.78**	0.65–0.94	0.75	0.55–1.01
Secondary	1.01	0.83–1.22	0.96	0.82–1.14	0.81	0.62–1.07
Tertiary (Ref)	1		1		1	
Employment status						
Working/Self-employed	0.76*	0.62–0.94	0.69***	0.57–0.84	0.69*	0.51–0.93
Homemaker/Unemployed/Student	0.72**	0.58–0.89	0.91	0.74–1.11	0.93	0.68–1.25
Retiree (Ref)	1		1		1	
Marital status						
Never married	1.26**	1.08–1.48	0.43***	0.33–0.55	1.02	0.67–1.55
Widowed/Divorced/Separated	1.28**	1.09–1.49	0.90	0.77–1.05	0.90	0.71–1.13
Married (Ref)	1		1		1	
Household monthly income (MYR)						
Bottom 40% (B40)	1.12	0.92–1.37	1.12	0.90–1.40	1.93*	1.17–3.19
Middle 40% (M40)	0.99	0.79–1.23	1.04	0.82–1.34	1.57	0.91–2.69
Top 20% (T20) (Ref)	1		1		1	

Abbreviations: *Ref* Reference, *OR *Odds ratio, *95% CI *95% Confidence interval, *MYR *Malaysian Ringgit

All estimates are computed considering the relatively healthy group (Class 1) as the reference category

*p<0.05, **p<0.01, ***p<0.001

The odds of being assigned to the musculoskeletal, mobility and sensory disorders group were increased by 3.64 times (95% CI = 2.85–4.65) in those with no education and also significantly increased in those with primary education (adjusted-OR = 1.90, 95% CI = 1.57–2.31) compared to those attending tertiary education (Table 4). In contrast, having no formal education (adjusted-OR = 0.69, 95% CI = 0.51–0.95) and primary education level (adjusted-OR = 0.78, 95% CI = 0.65–0.94) have a significantly lower likelihood of belonging to the cardiometabolic group. No formal education attainment was associated with an increased odds of being assigned to the complex multimorbidity group (adjusted-OR = 1.60, 95% CI = 1.06–2.40). The odds of being assigned to the musculoskeletal, mobility and sensory disorders group were lower for working/self-employed participants (adjusted-OR = 0.76, 95% CI = 0.62–0.94) and homemakers/unemployed participants/students (adjusted-OR = 0.72, 95% CI = 0.58–0.89), relative to the retirees. The working/self-employed participants were also significantly and equivalently 0.69 times less likely to be assigned to the cardiometabolic group (95% CI = 0.57–0.84) and the complex multimorbidity group (95% CI = 0.51–0.93) compared to the relatively healthy group. Participants who have never married (adjusted-OR = 1.26, 95% CI = 1.08–1.48), as well as those who were widowed/divorced/separated (adjusted-OR = 1.28, 95% CI = 1.09–1.49), have nearly 30% higher odds of being assigned to the musculoskeletal, mobility and sensory disorders group compared to married individuals. While participants who were single or had never married had significantly lower odds of having cardiometabolic diseases compared to married individuals (adjusted-OR = 0.43, 95% CI = 0.33–0.55). Additionally, those with the lowest household monthly income (bottom 40% income) were more likely to be in the complex multimorbidity group (adjusted-OR = 1.93, 95% CI = 1.17–3.19).

Furthermore, we conducted an additional multinomial logistic regression incorporating the same set of socio-demographic factors and newly classified age subgroups (18–34, 35–59 and ≥ 60 years) to assess whether all the age groups were associated with the multimorbidity latent classes. This model was adjusted for the number of chronic conditions (see Supplementary Table S1). The results reaffirmed our findings, showing that older age consistently increased the likelihood of being assigned to the musculoskeletal, mobility and sensory disorders group, the cardiometabolic group and the complex multimorbidity group, in comparison to the relatively healthy group. This association was evident across all age categories, further underscoring the robustness of our results. The number of chronic conditions was highly associated with the latent classes, particularly the complex multimorbidity group. However, even after adjusting for the number of conditions, the strong relationships between the risk factors and the latent classes persisted, with all parameters of interest remaining statistically significant.

#### Multimorbidity patterns and mortality incidence

**Fig. 4:**
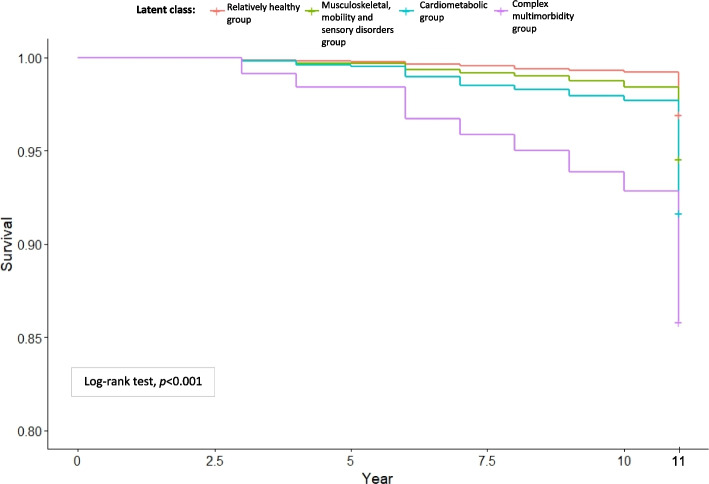
Kaplan–Meier survival curves for all-cause mortality by multimorbidity patterns

Figure 4 displays the probability of being alive across the multimorbidity latent classes over a period of 11 years. During that time period, 754 deaths (4.7%) were reported. Overall, the Kaplan–Meier survival curves by the latent classes showed that the four groups significantly differed in survival occurrence (log-rank test, *p* < 0.001).

The HRs and 95% CIs for the unadjusted and adjusted Cox proportional hazards regression models investigating the association between the incidence of all-cause mortality and LCA-based multimorbidity patterns are tabulated in Table 5. Latent class 1 (relatively healthy group) was considered as the reference group. The HR of the musculoskeletal, mobility and sensory disorders group and the cardiometabolic group was 1.81 (95% CI 1.48–2.22) and 2.82 higher (95% CI 2.36–3.36), respectively, and the highest was among the complex multimorbidity group (HR 5.03, 95% CI 4.02–6.30).

**Table 5:** Cox proportional hazards regression models investigating all-cause mortality risk as a function of multimorbidity patterns

Parameter	Unadjusted-model (*n* = 16,158)		Adjusted-model^a^ (*n* = 15,647)	
	HR	95% CI	HR	95% CI
Multimorbidity latent class				
Relatively healthy group (Ref)	1		1	
Musculoskeletal, mobility and sensory disorders group	1.81***	1.48–2.22	1.29*	1.04–1.59
Cardiometabolic group	2.82***	2.36–3.36	1.42***	1.18–1.70
Complex multimorbidity group	5.03***	4.02–6.30	1.83***	1.44–2.33

Abbreviations: *Ref* Reference, *HR *Hazard ratio, *95% CI *95% Confidence interval

^a^Cox proportional hazards regression models fitted were adjusted for age, sex, ethnicity, education and employment status

*p<0.05, **p<0.01, ***p<0.001

In the adjusted model controlling for baseline covariates (age, sex, ethnicity, education and employment status), the HRs for the three groups decreased, implying there is substantial confounding, with age and sex especially relevant (full estimates for the covariates are embedded in Supplementary Table S2). The HR for the musculoskeletal, mobility and sensory disorders group became least significant when adjusted for age and other covariates. The hazard of death among the cardiometabolic group and complex multimorbidity group were reduced in the adjusted model, but remained highly significant compared to the relatively healthy group (Table 5).

## Discussion

In the present study, we identified four distinct multimorbidity latent classes among a large sample of community-dwelling adults aged 18 and older and determined how these latent classes are associated with 11-year all-cause mortality, risk factors and HRQoL. To our knowledge, this is the first multimorbidity latent class cohort study of the general population in Malaysia and South East Asia. We used LCA to reduce the complexity of data and identify the multimorbidity subgroups, with a rigorous statistical approach that employs probability-based classification methods, enabling the selection of the most optimal number of classes of chronic conditions based on various diagnostic tests [28] and clinical meaning of the resulting multimorbidity patterns. There are several important learning points highlighted by our study findings. First, the multimorbidity subgroups identified are distinct. The multimorbidity patterns identified were musculoskeletal, mobility and sensory disorders; cardiometabolic diseases; and complex multimorbidity, respectively, in this population. Secondly, there is clear variation in socio-demographic characteristics and the associated adverse health outcomes between the multimorbidity classes, indicating which group of individuals were more vulnerable to multimorbidity and the consequences. We found that age, sex, ethnicity, education level and employment status were associated with distinct multimorbidity patterns. Those in the complex multimorbidity group had a significantly lower HRQoL than other groups across all aspects studied, including physical health, psychological, social relationship and environment domains. Thirdly, the mortality risk differed greatly across the multimorbidity patterns. Compared with the relatively healthy group, the complex multimorbidity group exhibited the highest mortality risk even after adjustment of age and other confounders in the analysis, followed closely by the cardiometabolic group and the musculoskeletal, mobility and sensory disorders group. This also implies that the combined effects of the number of chronic conditions, along with the type and severity of those conditions experienced might be proportional to the mortality risk. Fourthly, our study contributes rigorous evidence about multimorbidity in a middle-income country, aligning with findings from previous HIC studies.

The four multimorbidity classes identified in our study reflected the complexity of multimorbidity measurement and conceptualization. It is difficult to compare our findings directly with previous studies given the considerable methodological differences including study settings, disease types and counts, socio-demographic factors and the statistical methods adopted. Nonetheless, the multimorbidity patterns we identified generally agree with prior work, although limited relevant studies are available in LMICs. Such similarities may indicate that chronic conditions aggregate as they share common aetiology and underlying risk factors. For instance, we found that cardiovascular diseases (i.e. heart disease and stroke in our study) and metabolic diseases (i.e. hypertension, diabetes and obesity in this study) were very likely to co-occur, in agreement with the clinical nature of the chronic conditions, coupled with evidence from other studies [29, 30]. Although the mechanisms behind the combination of arthritis, chronic pain, and physical mobility, hearing and vision problems in this study remain unclear, a similar trend appears among these impairments—all related to physical disability and mobility limitations, which deserve more attention in future studies. Previous studies conducting LCA to describe multimorbidity patterns [31–34] have yielded similar patterns as our findings. All studies reported a ‘relatively healthy’ latent class (or a ‘minimal disease’ class), which consisted primarily of individuals with ≤ 1 medical condition. Likewise, these studies also reported a complex multimorbidity class with high prevalence in chronic conditions of multiple organ systems. Of the patterns reported, cardiovascular, metabolic, musculoskeletal and respiratory conditions were present in most studies in various combinations (e.g. cardiometabolic class consistent with the pattern we obtained in the present study). Despite using varied datasets and including different numbers of chronic conditions, LCA produced similar results for many studies, showing that some conditions may be more likely than others to co-occur and cluster together. These clusters importantly suggest practical implications for a shift from individual-disease approaches to a more integrated and holistic approach to health service delivery, focusing on clusters of multiple long-term conditions, to potentially improve outcomes and reduce the burden on healthcare systems. Based on our multimorbidity patterns identified, further research to better understand how these diseases interact may be the next helpful step. On the other hand, it is notable that the depression symptom levels did not vary much across the multimorbidity classes. This may be due to the measurement of depressive symptoms using the DASS-21 instrument based on a short recall period of 1 week, a methodological shortcoming that potentially limits the robustness of this result yet an assessment unavoidable in large HDSS data collection.

Tackling social determinants of multimorbidity is necessary to redress the rising burden of multimorbidity in disadvantaged populations. We found significant variations in socio-demographic composition between the multimorbidity groups. Our findings add to existing knowledge about age as a consistent and strong correlate of multimorbidity, across all multimorbidity patterns. Being female, having a lower educational level and not working were also more likely to belong to most of the multimorbidity groups than the relatively healthy group, albeit the significance varied across classes. Individuals with lower education levels were often associated with physically demanding blue-collar jobs, likely leading to higher rates of musculoskeletal problems. Furthermore, lower education levels, frequently linked to lower socioeconomic status, can result in poorer health outcomes and limited access to healthcare, health information and preventive care, thereby increased the risk of complex multimorbidity. Although this may seem counterintuitive, those with higher education levels were likely to engage in sedentary white-collar jobs, which may explain the increased risk of cardiometabolic diseases. Employment provides structured routines, social interactions and better access to healthcare through employment benefits, contributing to better health outcomes among those engaged in employment or non-retired activities, which may explain the consistent findings across latent classes among the study population. In our study, marital status appears to impact participants’ health through social support mechanisms. Married individuals might have better social support, leading to better health outcomes and access to care and, hence, a lower risk of musculoskeletal, mobility and sensory disorders. Single individuals might be younger or experience less stress related to marital responsibilities, reducing the risk of cardiometabolic conditions. The identified risk factors may support better integration of comprehensive management of multimorbidity in interventions and resources for optimal population health and addressing health disparities, targeting older adults, those with lower education levels and lower-income groups. Future research warrants a focus on the underlying pathogenesis connecting these chronic health conditions and the shared risk factors.

Given the potential of multimorbidity to erode financial security and compromise self-care capacity through the burden and complexity of managing multiple long-term conditions, the impact of multimorbidity patterns on quality-of-life outcomes is important. It comes as no surprise that the complex multimorbidity group has the poorest HRQoL compared to other groups across all aspects studied, including physical health, psychological, social relationship and environmental domains. This highlights the substantial impact of the most complex combination of chronic conditions on various aspects of well-being—as diseases clustered and increased in severity, so did the reduced quality of life, functional impairment and deteriorated psychological well-being, social relationships and the environment, in line with other studies [35]. These findings shed light on useful implementation of integrated mental and physical healthcare, and the importance of facilitating development of tailored preventive interventions and treatment for multimorbidity, particularly targeting the earlier stages of diseases.

In the Cox proportional hazards regression models, mortality risk was significantly higher across the multimorbidity groups in reference to the relatively healthy group over 11 years. Interestingly, although individuals in the musculoskeletal, mobility and sensory disorders group had a greater prevalence of chronic conditions (arthritis, chronic pain, physical mobility problems, hearing impairment and vision impairment) than the relatively healthy group, they had the lowest mortality risk among the three disease groups despite adjusted for confounders. This is in concordance with previous studies carried out in a cohort of 7197 community-dwelling adults aged 65 years and older in the USA [31], which found comparable mortality risk between their osteoarticular group (comprised of individuals with arthritis and osteoporosis) and minimal disease group, when stratified by their participants’ frailty status. The complex multimorbidity group in the present study has the strongest magnitude of association with mortality. A plausible explanation is the interplay between age and the most complex treatment and self-management alongside poorer health status in the complex multimorbidity group. Despite the inclusion of younger participants (≥ 18 years old), our sample consisted mostly of older individuals among those with multimorbidity, and the complex multimorbidity group had the highest overall age profile. This underscores the importance of addressing and prioritizing multimorbidity in clinical care to mitigate mortality risk, prioritizing the complex multimorbidity cohort.

Our study shows that people with complex multimorbidity, characterized by multiple severe chronic conditions, specifically overlapping musculoskeletal, mobility and sensory disorders, and cardiometabolic diseases, experienced the poorest HRQoL and the strongest mortality risk. This is particularly novel from a clinical perspective as it highlights the compounded detrimental effects of these chronic conditions, emphasizing the need for targeted care plans and resource allocation. Clinicians can use these insights to develop interventions that address the unique needs of those people with the highest number and severity of these chronic conditions. Our findings can also guide healthcare policymakers in optimizing resource distribution to high-risk groups, particularly in LMICs, where resources are often limited and need to be used strategically. Our findings advocate for integrated care models tailored to the needs of those with complex multimorbidity that ensure early detection and preventive measures, such as lifestyle modifications, regular screenings and proactive management to prevent the progression to more severe health states, for effective care and improved quality of life. This study adds to the body of literature by elucidating latent classes of multimorbidity and the consequences in Malaysia and South East Asia, and providing practical insights for improving patient outcomes and informing healthcare strategies, particularly beneficial for those with the most relevant complex and severe disease profiles.

There are some important limitations. First, we used 13 self-reported physician-diagnosed chronic conditions commonly available in epidemiological and clinical studies for LCA. On the ground that specific multimorbidity patterns may be sensitive to the number of chronic conditions [36], chronic conditions not evaluated in this study and our inclusion of additional chronic conditions might have yielded different multimorbidity patterns. In our study, information about cancer was not available. Conversely, other highly prevalent conditions such as physical mobility problems, obesity and depressive symptoms were considered in our study, although they are commonly omitted in other research. Second, although misclassification of individuals assigned to each latent class is reasonable because multimorbidity class membership is determined based on predictive probability calculated from LCA, there may still be some degree of uncertainty associated with latent class membership in some cases. Even though each class seemed to show clinically distinct patterns of chronic conditions, results should be interpreted with caution. We have shared clinical interpretations for these latent class memberships; future research may help to provide further certainty about the nature of these latent classes. One possibility is to extend the analysis to populations in other South East Asian countries or beyond. Third, our data on chronic conditions did not allow us to assess changes in the patterns of multimorbidity over time. It was also not possible to draw temporal associations with socio-demographic factors due to the limitation of datasets. Lifestyle risk factors which may be associated with the development of multimorbidity could not be included; future investigation may enhance understanding and offer opportunities for intervention. Lastly, it should be acknowledged that the depressive symptoms assessed by the DASS-21 instrument are not clinical diagnoses of depression. This may explain the lack of variation in depressive symptoms across the LCA groups and potentially their limited informativeness for constructing multimorbidity.

The main strengths of our study included a relatively large sample size provided enough numbers for LCA (thus leaving out bias towards conditions of specific prevalence or problems with low count rates), diverse and multi-ethnic representation, and a wide range of diseases and socio-demographic covariates studied. Another key strength of the present study from the modelling perspective is the use of a more objective LCA algorithm based on a rigorous statistical basis. We were able to identify distinct latent classes of the chronic health conditions in a robust manner beyond chance that are strongly associated with multimorbidity. Furthermore, the use of our health and demographic surveillance system data drawn from community-dwelling individuals allowed capturing a large number of participants who are not restricted to certain pre-requisites, such as belonging to a specific facility (e.g. solely hospitalized patients) or a specific health insurance company. Moreover, our databases with extensive and regular follow-up and up-to-date longitudinal death data make it a powerful surveillance tool for studying mortality in relation to multimorbidity. Incorporating this temporal dimension, spanning up to 11 years of mortality data, enriched our analysis by allowing associations to be directional, thereby provided stronger evidence for causality. We have also explicitly included relevant factors particularly the socio-demographic characteristics, thus, allowed the isolation of the effect of the survivorship bias. Most previous studies were conducted among the elderly, where multimorbidity is more likely to occur; our study bridged this gap by providing crucial evidence on population-based multimorbidity patterns across a broad age span comprising young, middle-aged and older persons. Our findings support proposals that interventions to improve outcomes in multimorbidity may be more appropriately targeted on the specific clusters uncovered. Our findings also underline the need for tailored approaches for higher-risk patient groups with important differences in socio-demographic characteristics and mortality risk.

## Conclusions

This study contributes rigorous evidence about multimorbidity in a middle-income country in South East Asia, with similar findings to previous HIC studies. We have identified four main multimorbidity patterns that were statistically and clinically distinct: musculoskeletal, mobility and sensory disorders group, cardiometabolic group, complex multimorbidity group and a relatively healthy group. By establishing which chronic conditions tend to cluster together, healthcare professionals can implement a more structured care plan rather than a single-disease treatment regime for patients with multimorbidity. Individuals of the four multimorbidity classes exhibited different socio-demographic characteristics, survival rates and health-related quality of life. Our research suggests that the complex multimorbidity group faces a significantly heightened risk of mortality and the lowest quality of life. By identifying which disease group had the highest risk of associated detrimental health and social inequalities based on findings about risk factors of multimorbidity patterns, we could offer valuable insights into targeted multimorbidity prevention and management to reduce the burden on the vulnerable population and health system. Our findings emphasize the need for an integrated approach to healthcare, accounting for the clusters of multiple conditions and prioritizing the complex multimorbidity cohort. Further research, particularly longitudinal studies are warranted to understand the underlying mechanisms linking chronic conditions, evolution of multimorbidity patterns and their impact on health and wellbeing of individuals.

## Supplementary Information

Additional file 1: Tables S1 – S2. Table S1: Multinomial logistic regression modelling results of factors associated with multimorbidity patterns, adjusted for number of chronic conditions. Table S2: Cox proportional hazards regression models (including estimates for the covariates) investigating all-cause mortality risk as a function of multimorbidity patterns.

## Data Availability

Data are available on request from the corresponding authors, or by application to the SEACO, Jeffrey Cheah School of Medicine and Health Sciences, Monash University, Malaysia.

## Abbreviations

Adjusted-ORs: Adjusted odds ratios
ANCOVA: Analysis of covariance
ANOVA: One-way analysis of variance
BIC: Bayesian–Schwarz Information Criterion
BMI: Body mass index
CAIC: Consistent Akaike Information Criterion
CI: Confidence interval
DASS-21: Depression, Anxiety and Stress Scale–21
DM: Diabetes mellitus
EFA: Exploratory factor analysis
ESRF: End-stage renal failure
HDSS: Health and demographic surveillance system
HICs: High-income countries
HR: Hazard ratio
HRQoL: Health-related quality of life
LCA: Latent class analysis
LMICs: Low- and middle-income countries
MRC: Medical Research Council
MUHREC: Monash University Human Research Ethics Committee
MUTUAL: MUltimorbidity ThroUgh cApacity buiLding
MYR: Malaysian Ringgit
NIHR: National Institute for Health and Care Research
OR: Odds ratio
Ref: Reference
SD: Standard deviation
SEACO: South East Asia Community Observatory
UKRI: UK Research and Innovation
WHO: World Health Organization
WHOQOL-BREF: World Health Organization Quality of Life Brief Version
