# Supplementary material for: Multimorbidity latent classes in relation to 11-year mortality, risk factors and health-related quality of life in Malaysia: a prospective health and demographic surveillance system study
Source: BMC Med. 2025 Jan 6;23:5. doi: 10.1186/s12916-024-03796-z (PMC11702131; doi:10.1186/s12916-024-03796-z)
Supplement: Supplementary file 1 — Additional file 1: Tables S1 – S2. Table S1: Multinomial logistic regression modelling results of factors associated with multimorbidity patterns, adjusted for number of chronic conditions. Table S2: Cox proportional hazards regression models (including estimates for the covariates) investigating all-cause mortality risk as a function of multimorbidity patterns. [file 12916_2024_3796_MOESM1_ESM.docx]

**Supplementary Information**

**Additional file 1:**

**Table S1** Multinomial logistic regression modelling results of factors associated with multimorbidity patterns, adjusted for number of chronic conditions

| **Latent Class** | | | | | | |
| --- | --- | --- | --- | --- | --- | --- |
| **Parameter** | **Musculoskeletal, mobility and sensory disorders group** | | **Cardiometabolic group** | | **Complex multimorbidity group** | |
|  | **Adjusted-OR** | **95% CI** | **Adjusted-OR** | **95% CI** | **Adjusted-OR** | **95% CI** |
| Age group (years)  18-34  35-59  60 and above (Ref) | 0.47***  0.50***  1 | 0.38–0.59  0.43–0.58 | 0.10***  0.49***  1 | 0.07–0.14  0.42–0.57 | 0.06***  0.25***  1 | 0.03–0.13  0.19–0.33 |
| Sex  Male  Female (Ref) | 0.93  1 | 0.80–1.08 | 1.24*  1 | 1.05–1.47 | 1.37*  1 | 1.01–1.85 |
| Ethnicity  Malay  Chinese  Indian (Ref) | 1.21  1.54***  1 | 0.98–1.49  1.23–1.95 | 0.95  1.05  1 | 0.77–1.18  0.83–1.34 | 1.09  1.27  1 | 0.71–1.67  0.79–2.03 |
| Education  No formal education  Primary  Secondary  Tertiary (Ref) | 2.88***  1.90***  0.99  1 | 2.11–3.95  1.50–2.42  0.78–1.25 | 0.56**  0.82  0.95  1 | 0.38–0.82  0.65–1.05  0.76–1.18 | 1.61  0.79  0.80  1 | 0.88–2.96  0.50–1.25  0.53–1.22 |
| Employment status  Working/  Self-employed  Homemaker/  Unemployed/  Student  Retiree (Ref) | 0.86  0.66**  1 | 0.66–1.12  0.51–0.87 | 0.68**  0.80  1 | 0.53–0.88  0.61–1.05 | 0.53**  0.45***  1 | 0.34–0.82  0.29–0.70 |
| Marital status  Never married  Widowed/Divorced/Separated  Married (Ref) | 1.10  1.25*  1 | 0.90–1.36  1.02–1.52 | 0.52***  1.06  1 | 0.38–0.71  0.87–1.30 | 0.98  1.17  1 | 0.53–1.80  0.83–1.65 |
| Household monthly income (MYR)  Bottom 40% (B40)  Middle 40% (M40)  Top 20% (T20) (Ref) | 1.38*  1.08  1 | 1.07–1.78  0.81–1.42 | 1.38*  1.04  1 | 1.04–1.83  0.76–1.43 | 2.33*  1.18  1 | 1.19–4.55  0.57­–2.45 |
| Number of chronic conditions | 8.40*** | 7.76–9.10 | 10.48*** | 9.64–11.40 | 53.30*** | 46.10–61.64 |

*Abbreviation:* Ref=Reference; OR=Odds ratio; 95% CI=95% Confidence interval; MYR=Malaysian Ringgit.

All estimates are computed considering the relatively healthy group (Class 1) as the reference category.

**p*<0.05, ***p*<0.01, ****p*<0.001

**Table S2** Cox proportional hazards regression models (including estimates for the covariates) investigating all-cause mortality risk as a function of multimorbidity patterns

| **Parameter** | **Unadjusted-model**  **(n=16,158)** | | **Adjusted-model**  **(n=15,647)** | |
| --- | --- | --- | --- | --- |
|  | **HR** | **95% CI** | **HR** | **95% CI** |
| Multimorbidity latent class  Relatively healthy group (Ref)  Musculoskeletal, mobility and sensory  disorders group  Cardiometabolic group  Complex multimorbidity group | 1  1.81***  2.82***  5.03*** | 1.48–2.22  2.36–3.36  4.02–6.30 | 1  1.29*  1.42***  1.83*** | 1.04–1.59  1.18–1.70  1.44–2.33 |
| Age |  |  | 1.07*** | 1.06–1.08 |
| Sex  Male (Ref)  Female |  |  | 1  0.42*** | 0.35–0.50 |
| Ethnicity  Malay (Ref)  Chinese  Indian |  |  | 1  0.98  1.00 | 0.81–1.18  0.78–1.27 |
| Education  No formal education (Ref)  Primary  Secondary  Tertiary |  |  | 1  1.26  1.89**  2.48*** | 0.80–1.97  1.23–2.91  1.56–3.96 |
| Employment status  Working/Self-employed (Ref)  Homemaker/Unemployed/Student  Retiree |  |  | 1  1.28*  0.75* | 1.05–1.56  0.58–0.98 |

*Abbreviation:* Ref=Reference; HR=Hazard ratio; 95% CI=95% Confidence interval.

**p*<0.05, ***p*<0.01, ****p*<0.001
